# Quantifying the patient´s perspective in neuromyelitis optica spectrum disorder: Psychometric properties of the SymptoMScreen questionnaire

**DOI:** 10.1371/journal.pone.0255317

**Published:** 2021-07-29

**Authors:** José E. Meca-Lallana, Jorge Maurino, Francisco Pérez-Miralles, Lucía Forero, María Sepúlveda, Carmen Calles, María L. Martínez-Ginés, Inés González-Suárez, Sabas Boyero, Lucía Romero-Pinel, Ángel P. Sempere, Virginia Meca-Lallana, Luis Querol, Lucienne Costa-Frossard, Daniel Prefasi, Rocío Gómez-Ballesteros, Javier Ballesteros

**Affiliations:** 1 Department of Neurology, Clinical Neuroimmunology Unit and Multiple Sclerosis CSUR, Hospital Universitario “Virgen de la Arrixaca”, IMIB-Arrixaca, Murcia, Spain; 2 Medical Department, Roche Farma, Madrid, Spain; 3 Department of Neurology, Unit of Neuroimmunology, Hospital Universitari i Politècnic La Fe, Valencia, Spain; 4 Department of Neurology, Hospital Universitario Puerta del Mar, Cádiz, Spain; 5 Department of Neurology, Hospital Clínic i Provincial de Barcelona, Barcelona, Spain; 6 Department of Neurology, Hospital Universitari Son Espases, Palma de Mallorca, Spain; 7 Department of Neurology, Hospital Universitario Gregorio Marañón, Madrid, Spain; 8 Department of Neurology, Hospital Universitario Álvaro Cunqueiro, Vigo, Spain; 9 Department of Neurology, Hospital Universitario Cruces, Bilbao, Spain; 10 Department of Neurology, Hospital Universitari de Bellvitge, Barcelona, Spain; 11 Department of Neurology, Hospital General Universitario de Alicante, Alicante, Spain; 12 Department of Neurology, Hospital Universitario La Princesa, Madrid, Spain; 13 Department of Neurology, Hospital de la Santa Creu i Sant Pau, Barcelona, Spain; 14 Department of Neurology, Hospital Universitario Ramón y Cajal, Madrid, Spain; 15 Department of Neurosciences and CIBERSAM, University of Basque Country (UPV/EHU), Leioa, Spain; 16 Biocruces Bizkaia Health Research Institute, Barakaldo, Spain; Universita degli Studi di Napoli Federico II, ITALY

## Abstract

**Background:**

The assessment of self-reported outcomes in neuromyelitis optica spectrum disorder (NMOSD) is limited by the lack of validated disease-specific measures. The SymptoMScreen (SyMS) is a patient-reported questionnaire for measuring symptom severity in different domains affected by multiple sclerosis (MS), but has not been thoroughly evaluated in NMOSD. The aim of this study was to assess the psychometric properties of the SyMS in a sample of patients with NMOSD.

**Methods:**

A non-interventional, cross-sectional study in adult subjects with NMOSD (Wingerchuk 2015 criteria) was conducted at 13 neuroimmunology clinics applying the SyMS. A non-parametric item response theory procedure, Mokken analysis, was performed to assess the underlying dimensional structure and scalability of items and overall questionnaire. All analyses were performed with R (v4.0.3) using the mokken library.

**Results:**

A total of 70 patients were studied (mean age: 47.5 ± 15 years, 80% female, mean Expanded Disability Status Scale score: 3.0 [interquartile range 1.5, 4.5]). Symptom severity was low (median SyMS score: 19.0 [interquartile range 10.0, 32.0]). The SyMS showed a robust internal reliability (Cronbach’s alpha: 0.90 [95% confidence interval 0.86, 0.93]) and behaved as a unidimensional scale with all items showing scalability coefficients > 0.30. The overall SyMS scalability was 0.45 conforming to a medium scale according to Mokken’s criteria. Fatigue and body pain were the domains with the highest scalability coefficients. The SyMS was associated with disability (rho: 0.586), and physical and psychological quality of life (rho: 0.856 and 0.696, respectively).

**Conclusions:**

The SyMS shows appropriate psychometric characteristics and may constitute a valuable and easy-to-implement option to measure symptom severity in patients with NMOSD.

## Introduction

Neuromyelitis optica spectrum disorder (NMOSD) is a rare neuroinflammatory condition targeting primarily the spinal cord and optic nerve and associated with severe disability [1,2]. Fatigue, pain, visual loss, and motor impairment are the most prevalent symptoms [3–5]. Depression and other psychiatric comorbid conditions are also common in NMOSD affecting well-being and quality of life [6,7]. However, little is known about the subjective experience of patients living with NMOSD [4,8,9]. In addition, the assessment of patients´ perspectives is limited by the lack of validated, self-reported, NMOSD-specific measures [9].

NMOSD and multiple sclerosis (MS) are different neuroinflammatory disorders, but share a heterogeneous spectrum of symptoms [1,7,10]. The SymptoMScreen (SyMS) is a validated, patient-reported questionnaire for measuring symptom severity in key domains affected by MS, and thus we hypothesized that it might be also relevant to gather the impact of NMOSD from the patients’ perspective [11,12].

The aim of this study was to assess the psychometric properties of the SyMS questionnaire in a sample of NMOSD patients.

## Materials and methods

A non-interventional, cross-sectional study was conducted to assess the reliability, dimensional structure, and item characteristics of the SyMS in patients with NMOSD (PERSPECTIVES-NMO study). Eligibility criteria included age at least 18 years old and a diagnosis of NMOSD according to Wingerchuk 2015 criteria [13]. This study was conducted in accordance with the Good Clinical Practice Guidelines of the International Conference on Harmonisation and with the ethical principles of the Declaration of Helsinki and was approved by the investigational review board of Galicia (CEIm-G), Santiago de Compostela, Spain. Patients were consecutively recruited in the context of their follow-up visits at thirteen hospital-based neuroimmunology clinics between November 2019 and July 2020. All participants provided written informed consent.

### SymptoMScreen questionnaire

The SyMS assesses symptom severity across twelve neurologic domains: mobility, hand function/dexterity, spasticity and stiffness, pain, sensory symptoms, bladder control, fatigue, vision, dizziness, cognition, depression, and anxiety [11,12,14]. Each item is assessed on a seven-point Likert scale: 0 (not at all affected) to 6 (total limitation). The total score ranges from 0 to 72, with higher scores indicating more severe symptom endorsement.

This questionnaire was developed in English and has been translated into 19 languages. A paper format is available free for download at https://www.symptomscreen.org/. The original Spanish version of the SyMS was administered in this study only replacing the term MS by NMOSD in the first statement: *“Please circle one number that best describes how each NMOSD symptom has affected your everyday life activities“*.

### Statistical analysis

We analyzed the internal reliability of the SyMS with Cronbach’s alpha index. We assessed the underlying dimensional structure of the SyMS with a non-parametric item response theory procedure, Mokken analysis [15]. We also used Mokken analysis to assess the scalability of items and overall questionnaire. The scalability coefficients (*H*_*i*_) for each item indicate the discrimination and weight each item has on the latent variable or dimension it is purported to measure. We required an internal reliability equal or greater than 0.70 but lower than 0.95 to define acceptable reliability. We required scalability coefficients (*H*_*i*_) for each item and the overall scale (*H*), to be equal or greater than 0.30 to define an acceptable scale [15]. All analyses were done with R (v4.0.3) using the *mokken* library [16,17]. Associations between the SyMS and the Expanded Disability Status Scale (EDSS) and the 29-item Multiple Sclerosis Impact Scale (MSIS-29) were analyzed using Spearman’s rank correlation [18,19].

## Results

A total of 70 patients were studied. The mean (± SD) age was 47.5 ± 15 years and 80% were female. The median EDSS score was 3.0 (interquartile range 1.5, 4.5). Seven patients (10%) required a walking aid and three (4.3%) used a wheelchair. Eighteen patients (27.7%) had uni- or bilateral visual acuity lower than 20/100. Overall symptom severity was low with a median SyMS score of 19.0 (interquartile range 10.0, 32.0). Sociodemographic and clinical characteristics of the sample are shown in Table 1.

**Table 1:** Demographic and clinical characteristics.

	n = 70
Age (years), mean ± SD	47.5 ± 15
Sex (female), n (%)	56 (80)
Disease duration (years), mean ± SD	9.9 ± 8.1
Anti-aquaporin-4 antibodies, n (%)	54 (77.1%)
Relapsing form, n (%)	59 (84.2)
Single-attack form, n (%)	11 (15.7)
Number of relapses since diagnosis, mean ± SD	2.9 ± 2.3
Number of relapses in the last year, mean ± SD	0.5 ± 0.9
EDSS score, median (IQR)	3.0 (1.5, 4.5)
EDSS score ≥ 6, n (%)	10 (14.3)
SymptoMScreen score, median (IQR)	19.0 (10.0, 32.0)
Physical impact MSIS-29 score, mean ± SD	41.9 ± 16.7
Psychological impact MSIS-29 score, mean ± SD	20.9 ± 8.3

EDSS = Expanded Disability Status Scale; MSIS-29 = 29-item Multiple Sclerosis Impact Scale; IQR = interquartile range; SD = standard deviation.

**Fig 1:**
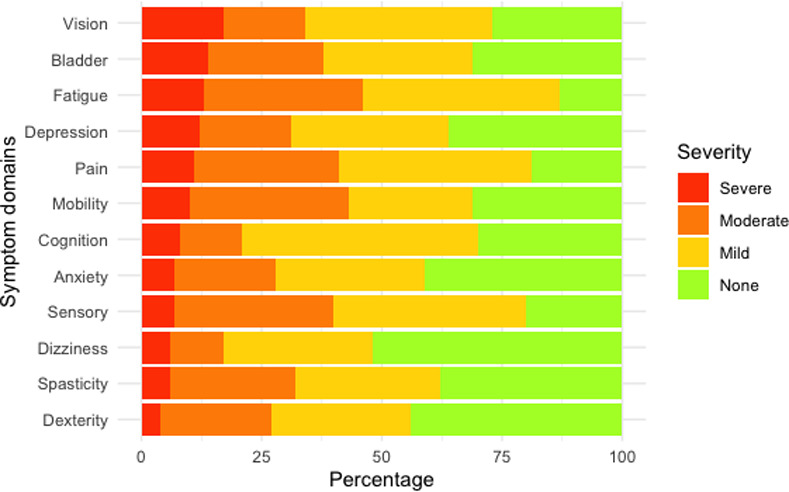
Symptom severity.

The SyMS showed a robust internal reliability (Cronbach’s alpha 0.90 [95% confidence interval 0.86, 0.93]) and behaved as a unidimensional scale with all items showing scalability coefficients *H*_*i*_ > 0.30. The overall SyMS scalability was 0.45 conforming to a medium scale according to Mokken’s criteria. Fatigue and body pain were the domains with the highest scalability coefficients, whereas vision and bladder control were the domains with the highest levels of perceived severity (Table 2 and Fig 1).

**Table 2:** Psychometric characteristics of the SymptoMScreen.

		Severity levels, n (%)			
Domain	*H*_*i*_	None (score 0)	Mild (score 1+2)	Moderate (score 3+4)	Severe (score 5+6)
Mobility	0.45	22 (31)	18 (26)	23 (33)	7 (10)
Dexterity	0.39	31 (44)	20 (29)	16 (23)	3 (4)
Spasticity	0.46	27 (39)	21 (30)	18 (26)	4 (6)
Pain	0.52	13 (19)	28 (40)	21 (30)	8 (11)
Sensory symptoms	0.49	14 (20)	28 (40)	23 (33)	5 (7)
Bladder control	0.39	21 (30)	22 (31)	17 (24)	10 (14)
Fatigue	0.53	9 (13)	29 (41)	23 (33)	9 (13)
Vision	0.36	19 (27)	27 (39)	12 (17)	12 (17)
Dizziness	0.43	36 (51)	22 (31)	8 (11)	4 (6)
Cognition	0.48	21 (30)	34 (49)	9 (13)	6 (9)
Depression	0.46	25 (36)	23 (33)	13 (19)	9 (13)
Anxiety	0.40	28 (40)	22 (31)	15 (21)	5 (7)

*H*_*i*_ = scalability coefficient.

Overall SyMS score showed a significant correlation with physical and psychological MSIS-29 scores (rho = 0.856 and 0.696, p<0.0001, respectively), and EDSS score (rho = 0.586, p<0.0001).

## Discussion

NMOSD is a rare autoimmune, neuroinflammatory disease with a negative impact on patients´ independence and quality of life [4,8,20]. The unpredictability trajectory of the disease makes the experience of living with NMOSD even more difficult for patients and their caregivers [7,8,21].

The assessment of patient perspectives using standardized self-report instruments have been inconsistently conducted in patients with neuroinflammatory and demyelinating disorders [9,22]. Well-designed and validated patient-oriented outcome measures in NMSOD are needed to better capture the course of the disease and the response to treatments [3,4,9]. The US online community PatientsLikeMe developed the Neuromyelitis Optica Rating Scale (NMORS), a self-reported, eight-item instrument to assess symptoms and disability in patients with NMOSD [3]. The total score ranges from 0 to 100, with higher scores indicating more severe symptom severity. Moore et al. created a 46-item patient-reported questionnaire to assess the impact of NMOSD on life functioning and different symptom dimensions, including vision, mobility, bladder, bowel, mood, sexual dysfunction, pain, fatigue, and cognition [9]. However, neither of the two instruments is psychometrically validated.

The SyMS is a validated, brief and easy-to-implement questionnaire to quantify symptoms in MS [11,12]. The overall score correlates with clinical disability, physical and psychological health-related quality of life, and work productivity [14,23]. Green et al. assessed symptom severity using the SyMS in a sample of 43 patients with NMOSD and 85 with MS [24]. Although overall scores were similar in both populations, vision impairment, spasticity, pain, and bladder dysfunction were worse in patients with NMOSD compared to MS.

Our study found that the SyMS is a patient-reported instrument with a high internal reliability and a unidimensional construct also in patients with NMOSD. These results enable its implementation in clinical practice to capture the impact of the disease in multiple relevant and clinically meaningful symptom domains. To our knowledge, this is the first study to assess psychometric issues of the SyMS questionnaire in NMOSD, and its results help to support its use and add to the scarce presence of patient-reported measures in NMOSD.

Less tangible symptoms, usually not detected on the standard neurological examination like fatigue, pain or cognitive problems may be often more important to patients’ sense of well-being and quality of life than physical disability in MS [25]. More than forty percent of 522 NMOSD patients participating in a recent survey reported moderate or severe disability due to fatigue and pain [3]. Cognitive impairment in NMOSD may already affect patients with a low level of physical disability [26]. A meta-analysis including 25 studies and 761 patients found a prevalence of cognitive impairment of 34% (95% CI:31–37%) [27]. Longer disease duration, fatigue, pain, and sleep disturbances were associated with cognitive impairment [26]. These “less overt impairments” stress the need for including patient-centered measures in clinical practice.

Our study population included a sample of clinically stable NMOSD patients with a medium degree of physical disability and mostly with positive anti-aquaporin-4 antibodies. The fact that most of the recruitment period coincided with the onset of the COVID-19 pandemic could influence the selection of patients with this profile, since the most disabled patients avoided conducting face-to-face consultations. The results may thus not be generalizable to subjects with less stable disease status or higher disability and other serologic subtypes. The small sample size due to a low prevalence of NMOSD did not allow us to perform a parametric confirmatory factor analysis and compare the fit of different solutions to data [28]. Further studies with a larger sample size are needed to confirm the unidimensional structure of the SyMS. Despite this limitation, the sample of 70 subjects was managed at 13 different clinics on a national level, representing NMOSD patients across the country.

## Conclusion

Although the SyMS is not a disease-specific measurement, it shows appropriate psychometric characteristics and may constitute a brief and reliable option to assess symptom severity in patients with NMOSD.
